# Supplementary material for: The origin of jerky dislocation motion in high-entropy alloys
Source: Nat Commun. 2022 Aug 15;13:4777. doi: 10.1038/s41467-022-32134-1 (PMC9378647; doi:10.1038/s41467-022-32134-1)
Supplement: Supplementary file 1 — Supplementary Information [file 41467_2022_32134_MOESM1_ESM.pdf]

Supplementary Materials for

The origin of jerky dislocation motion in high-entropy alloys

Daniel Utt, Subin Lee, Yaolong Xing, Hyejin Jeong, Alexander Stukowski, Sang Ho Oh,

Gerhard Dehm, Karsten Albe

Correspondence to: shoh@kentech.ac.kr, dehm@mpie.de, albe@mm.tu-darmstadt.de

Supplementary Figures

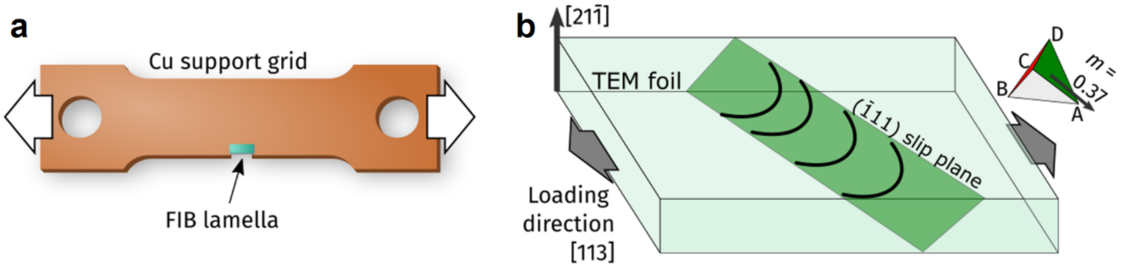

Supplementary Figure 1. **Geometry of in-situ TEM samples. a** Cu supporting grid for in-situ straining holder. A small notch is cut by FIB milling, and the FIB lamella was positioned on top of it. **b** A schematic drawing showing crystallographic orientation of the sample. Uniaxial load which was directly applied to the Cu inset, also strained the TEM foil. Dislocations in Fig.1 **a** glided on the $(\bar{1}11)$ plane which was inclined with respect to the electron beam direction.

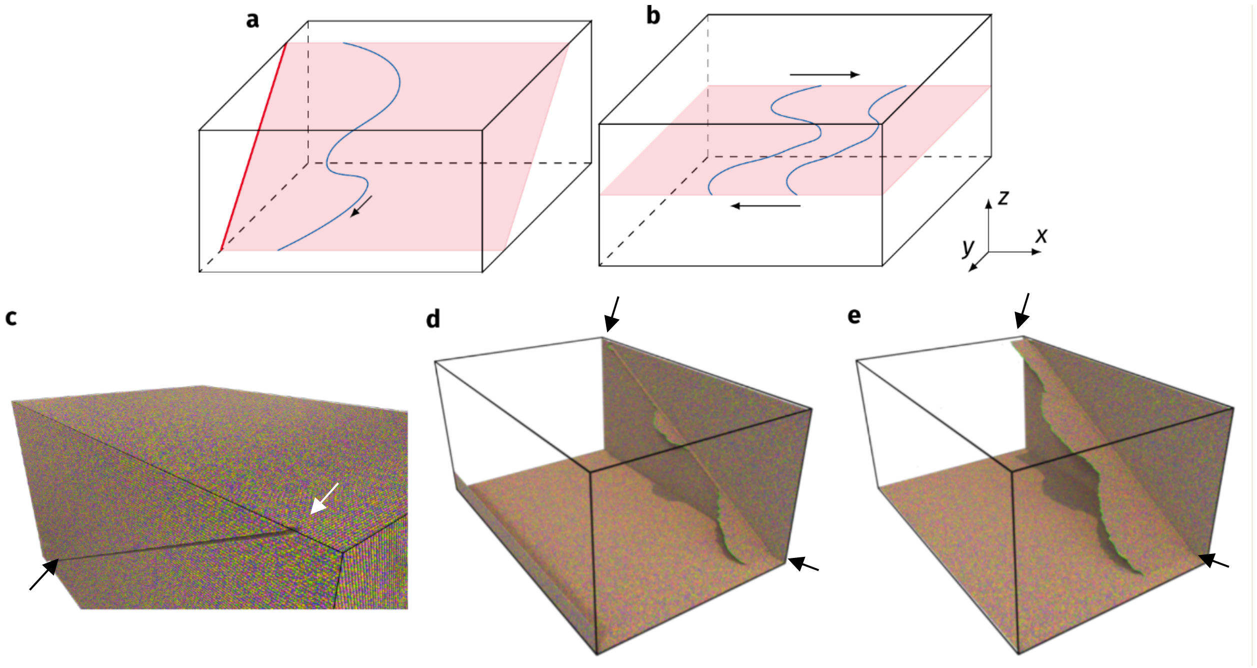

Supplementary Figure 2. **Schematic drawing of the samples used the atomistic computer simulations. a** Simulated TEM lamella used to investigate the dislocation nucleation from a surface notch (red line). Shockley partial dislocations (blue line) nucleate under the applied tensile strain (arrow) and move on the {111} glide plane (red). The sample has periodic boundary conditions along the *y*-direction with open boundaries along *x*- and *z*-directions. **b** Sample setup used to determine the critical force F_C for dislocation glide and the dislocation mobility *M*. Shockley partial dislocations (blue) adding up to a perfect edge dislocation are inserted on a {111} glide plane (red). They start to glide under the applied shear force on the top and bottom surface layers (arrows). The sample has open boundaries along *z*-direction, while the two other directions have periodic boundary conditions. Further details are given in the methodology section. **c** Close-up of the notch cut into the large CoCrFeMnNi sample after equilibration at 5 K for 50 ps prior to straining. **d-e** Dislocation nucleating from the notch under applied strain. The dislocation line determined from Dislocation analysis (DXA), implemented in OVITO is shown in green, non-FCC atoms are hidden for better visibility of the leading partial dislocation and resulting stacking fault (SF). Atoms on the front and top of the sample are hidden as well to allow for a view inside the sample. The position of the notch is indicated by arrows.

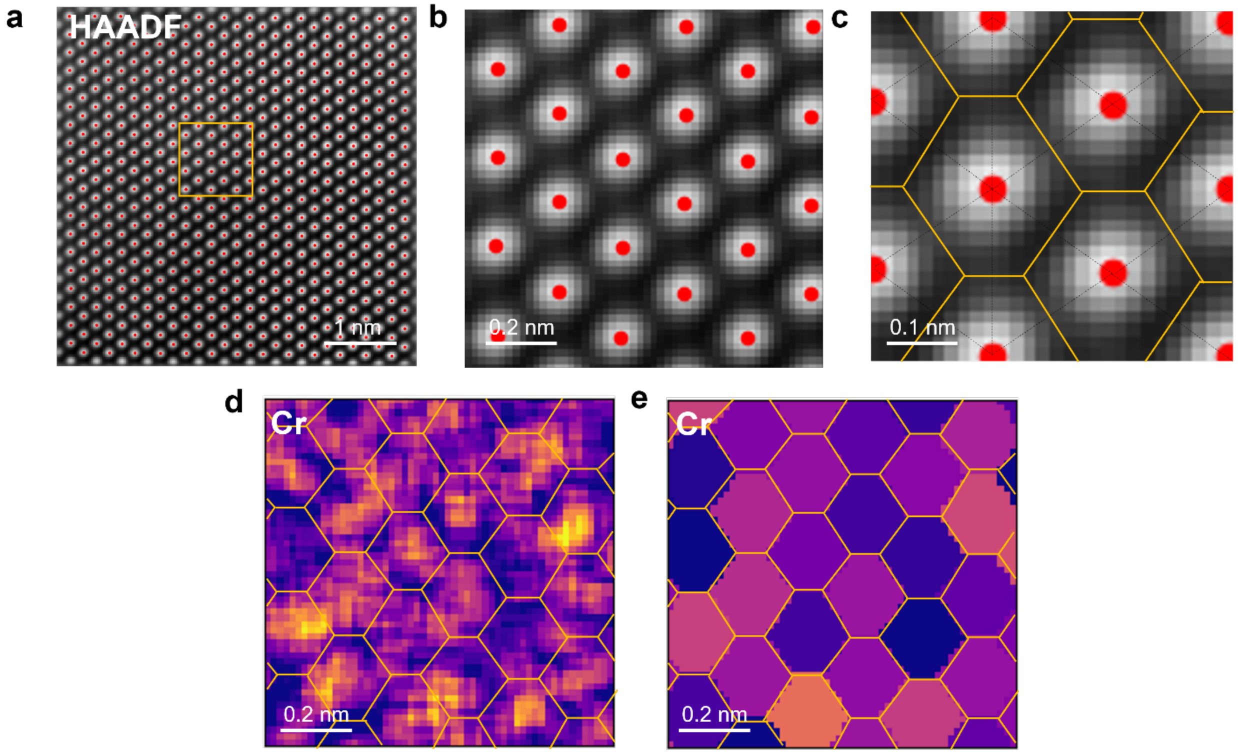

Supplementary Figure 3. **Voronoi cell representation of EDS composition map. a** STEM HAADF image recorded in [110] zone axis. **b** Magnified STEM HAADF image showing the position (red dot) of atomic columns determined by Gaussian fitting of the column intensity. **c** Voronoi cells constructed based on the atomic column position. **d** Voronoi cells overlaid on EDS concentration map of Cr. The (5×5) binning was applied to the original pixels of EDS map. **e** EDS composition map generated by averaging the data within each Voronoi cell.

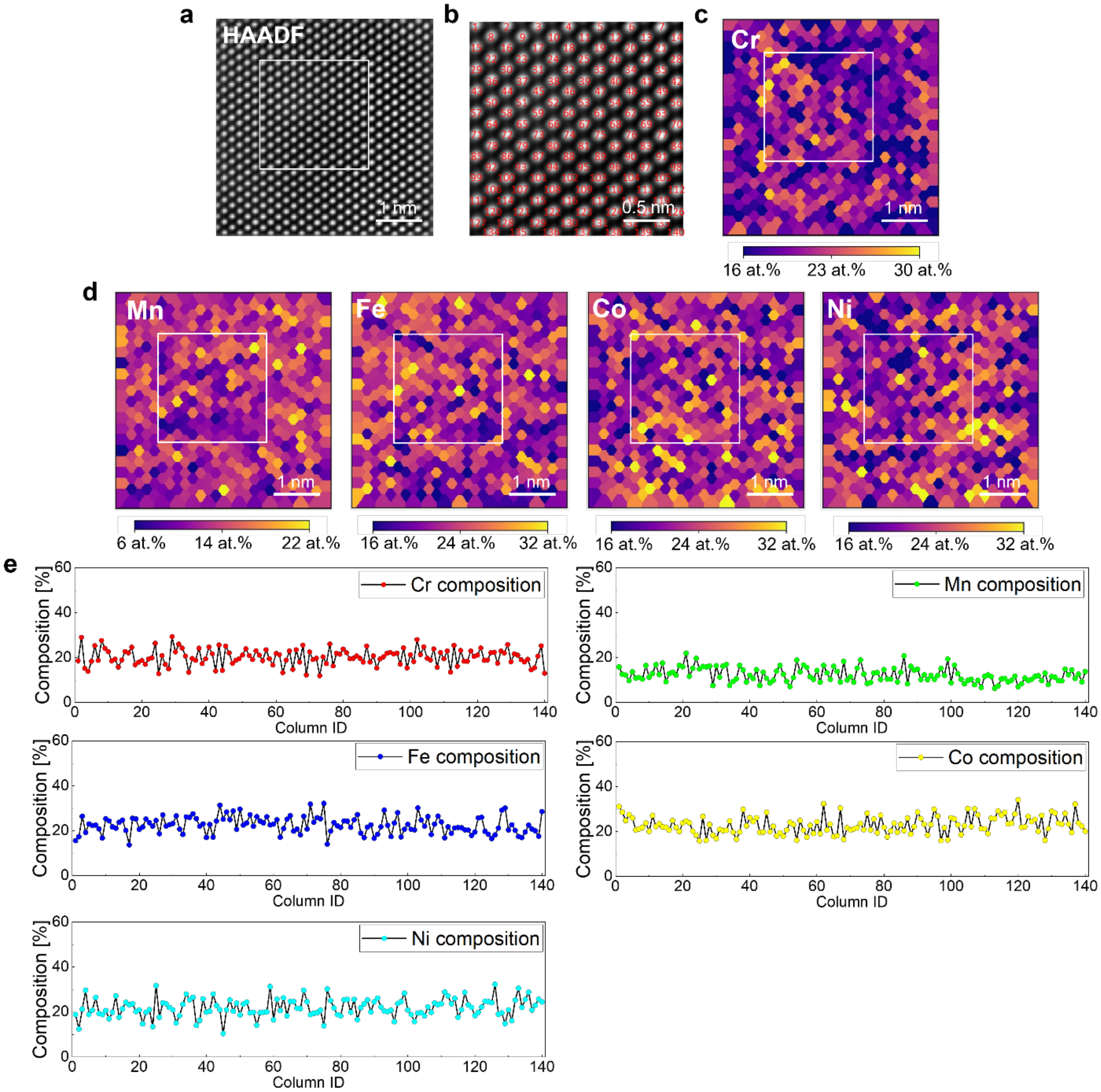

Supplementary Figure 4. **EDS composition maps of dislocation-free region. a** STEM HAADF image recorded in [110] zone axis. **b** Magnified view of STEM HAADF image (white-lined box in **a**) showing the atomic columns indexed for composition plots in **c-e**. EDS composition map of **c** Cr and **d** all the other elements. **e** EDS composition plot of each element within the white-lined box. The column ID is defined in **b**. Each element shows random fluctuation without noticeable clustering.

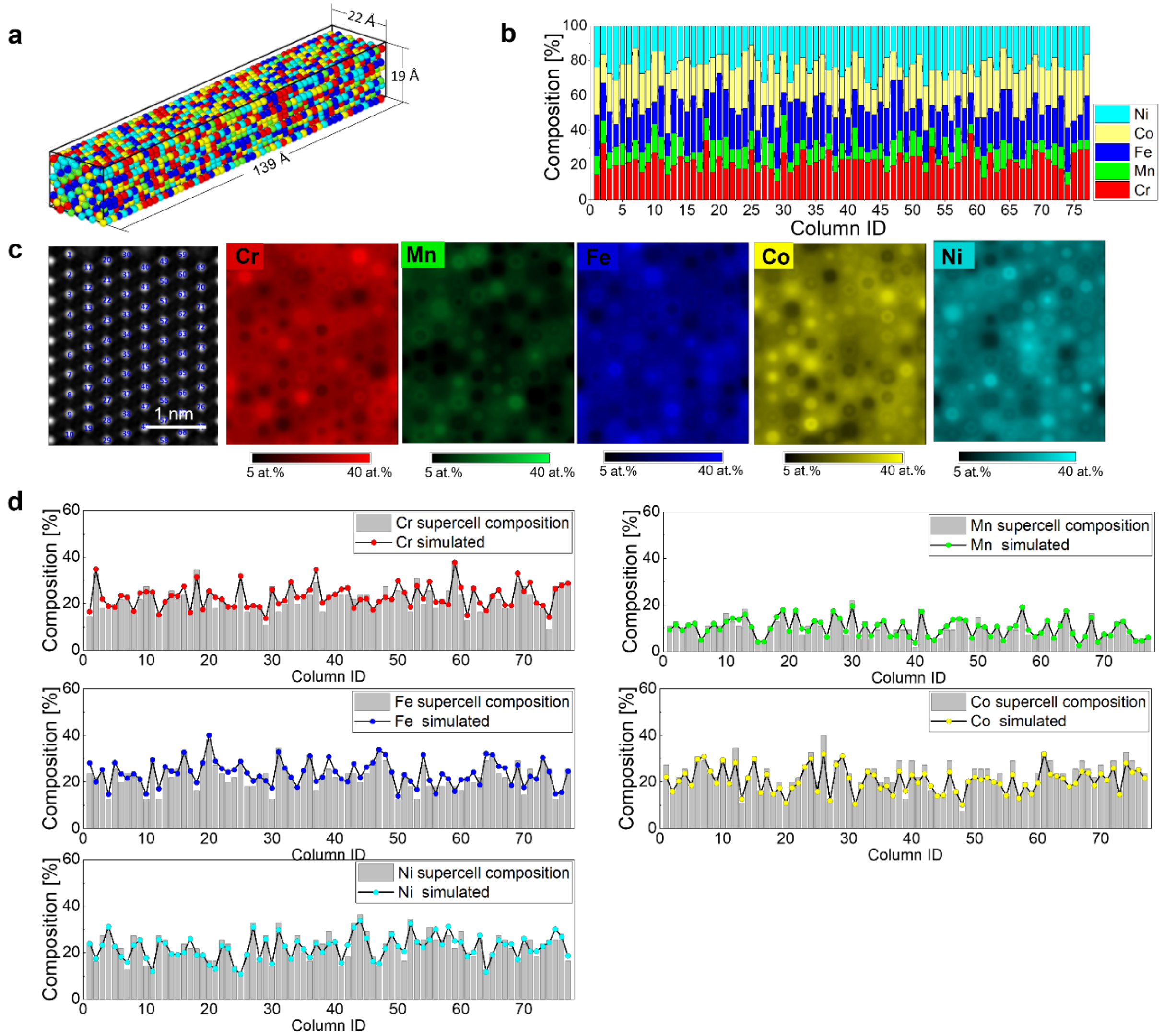

Supplementary Figure 5. **Simulated EDS composition maps of a randomly configured HEA. a** Atomic simulation cell. **b** Plot showing the composition of each atomic column in the simulation cell. **c** Simulated HAADF image and EDS composition maps using µSTEM (version 4.5). In the STEM HAADF image the atomic columns are indexed, starting with position 1 on the top left and ending with position 77 at the lower right, for composition plots in **d**. **d** Simulated EDS composition (colored symbol and line) and the composition of simulation cell (gray bar) plotted for the atomic columns defined in **c**. The two data show almost perfect coincidence.

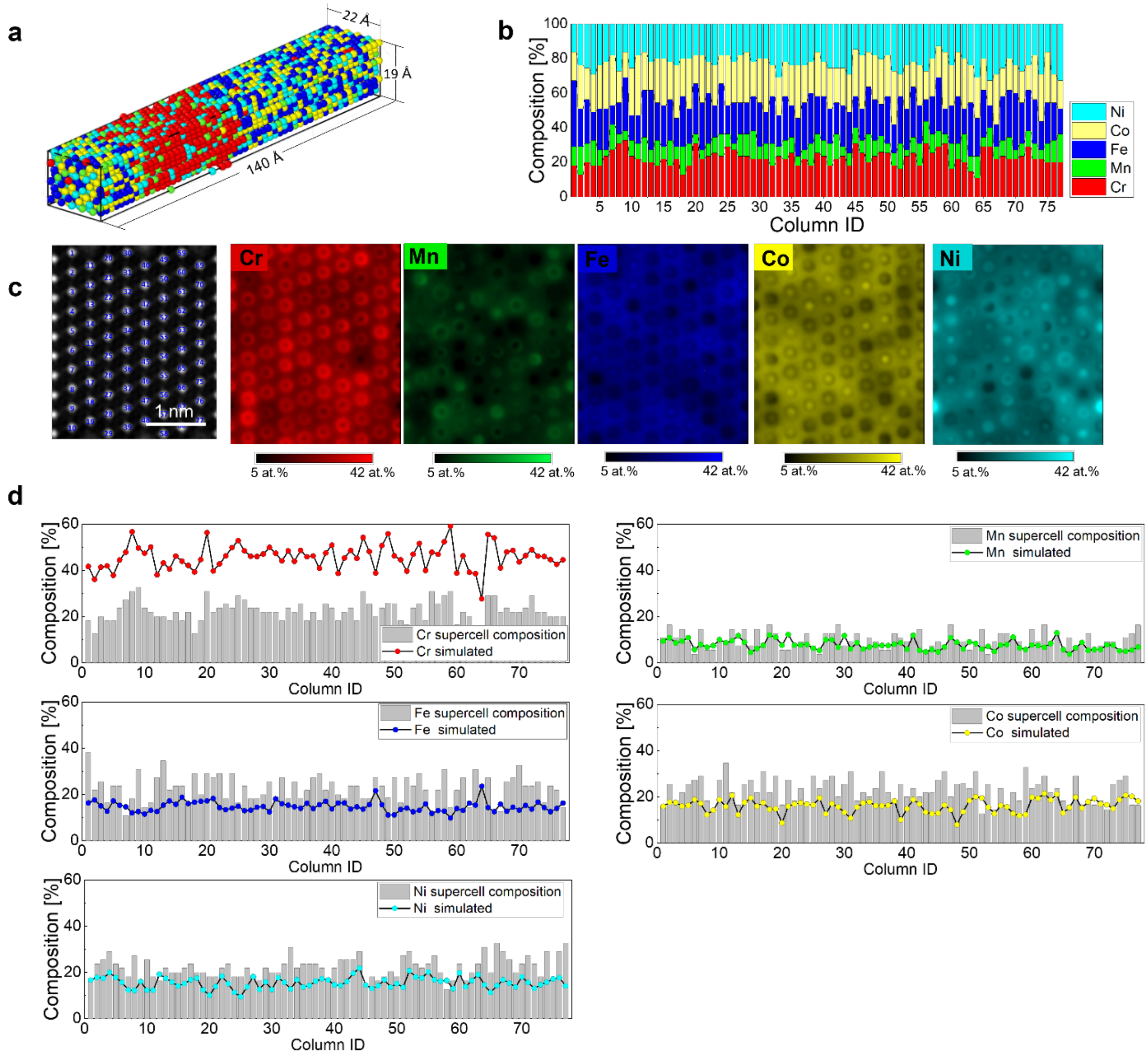

Supplementary Figure 6. **Simulated EDS composition maps of HEA with partially ordered Cr. a** Atomic simulation cell. **b** Plot showing the composition of each atomic column in the simulation cell. Note that the concentration of all elements remained the same as in the randomly configured alloy used for Fig. R1 but only their spatial distribution has been altered. **c** Simulated HAADF image and EDS composition maps. In the STEM HAADF image the atomic columns are indexed from 1 (top left) to 77 (bottom right) for composition plots in **d**. **d** Simulated EDS composition (colored symbol and line) and the composition of simulation cell (gray bar) plotted for the atomic columns defined in **c**. The composition of the partially ordered element Cr is overestimated by a factor of two whereas that of other elements is slightly underestimated due to the channeling effects on X-ray generation.

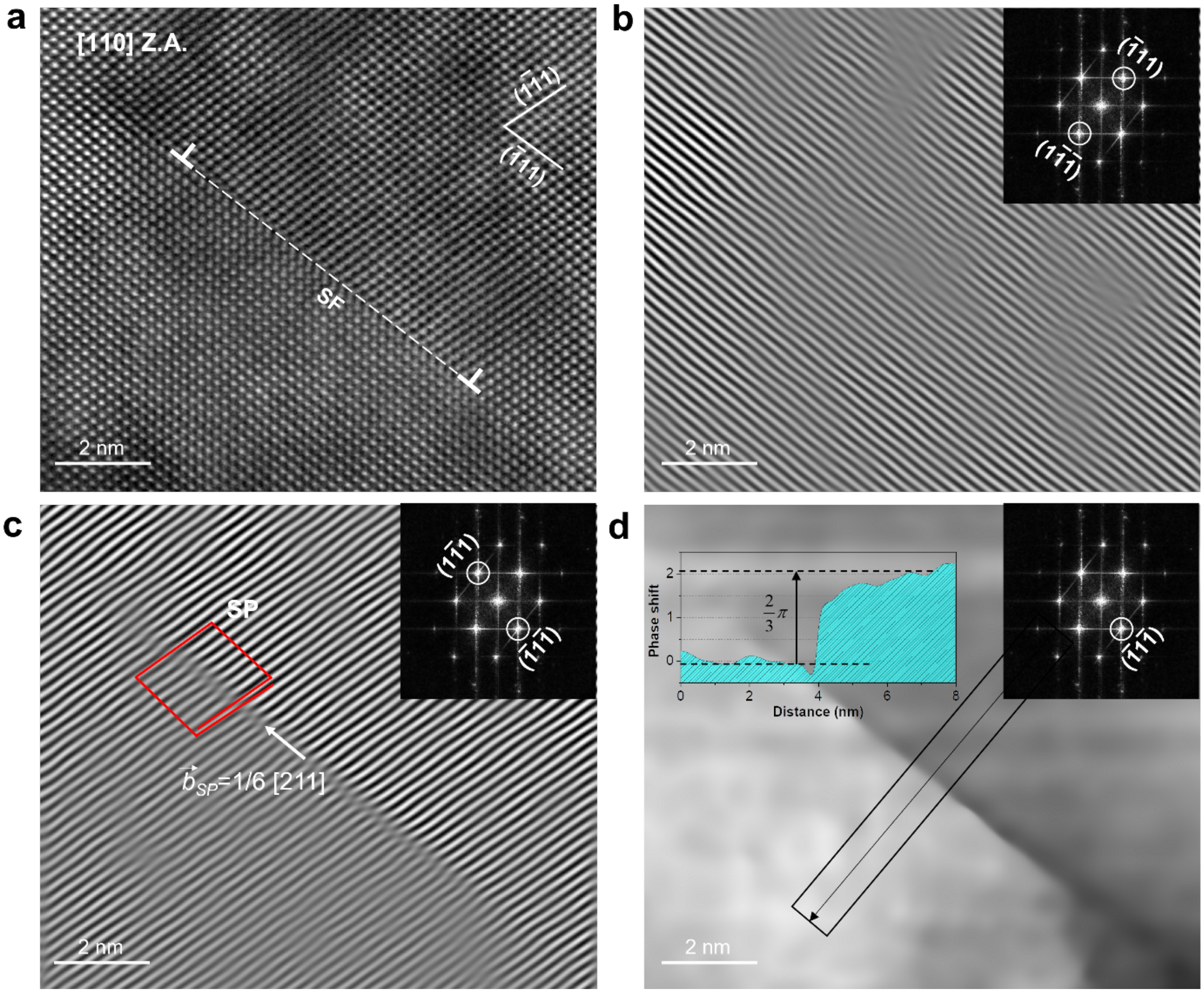

Supplementary Figure 7. **Analysis of the Burgers vector of Shockley partial dislocation. a** STEM HAADF image showing an extended dislocation consisted of Shockley partial (SP) dislocations and SF. **b** Bragg filtered image formed by selecting $(\bar{1}11)$ and $(1\bar{1}\bar{1})$ reflections. **c** Bragg filtered image formed by selecting $(1\bar{1}1)$ and $(\bar{1}1\bar{1})$ reflections. A Burgers circuit is drawn on SP, showing the Burgers vector of $a_0/6[211]$. **d** Geometric phase image obtained by GPA showing a phase shift of $2\pi/3$ upon crossing the SF.

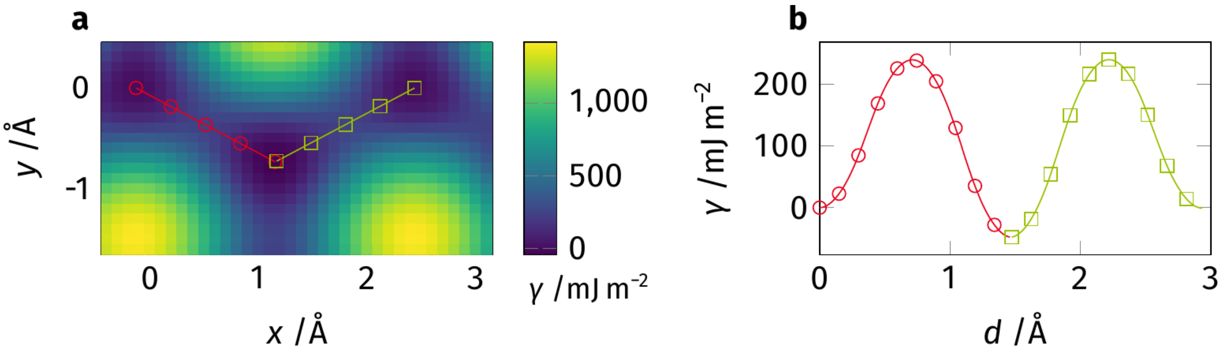

Supplementary Figure 8. **Generalized stacking fault curve of the equimolar Cantor alloy. a** GSF energy landscape for the leading (red) and trailing (green) partial dislocations' displacement in the Cantor alloy sample. Averaging is done over the whole glide plane. The local (FCC) and global (HCP) minima configuration are connected via linear displacements to obtain the energy landscape given in **b**.

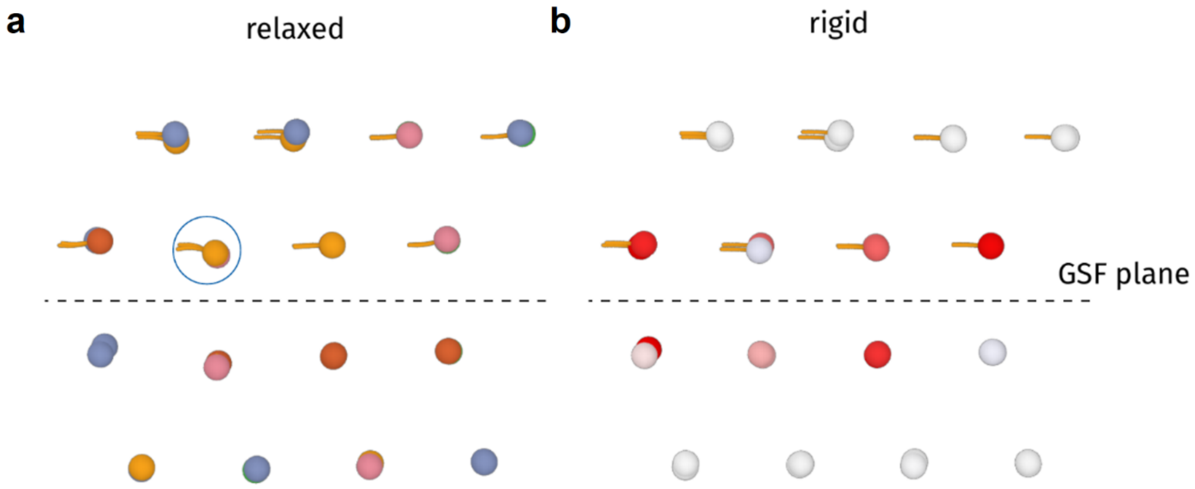

Supplementary Figure 9. **Comparison of the conventional and the rigid relaxation method.** Comparison of the two different SF calculation relaxation methods. Both snapshots show the saddle point configuration which corresponds to the unstable SF. Yellow lines show the full atomic trajectory starting from the ideal FCC configuration. **a** Standard methodology allowing for atomic relaxation normal to the fault plane. An atom with strong structural relaxation is highlighted. The atoms are color coded based on the species and the trajectory lines reveal atomic relaxations. **b** Proposed method, where the atoms are only allowed to follow rigid body relaxations. The atoms are color coded based on their atomic energy change during this calculation (red corresponds to an energy increase) which is subsequently used to calculate the atomic generalized stacking fault (GSF) energy curves (Fig. 3 **a**, **b**). Here, all trajectory lines are exactly parallel.

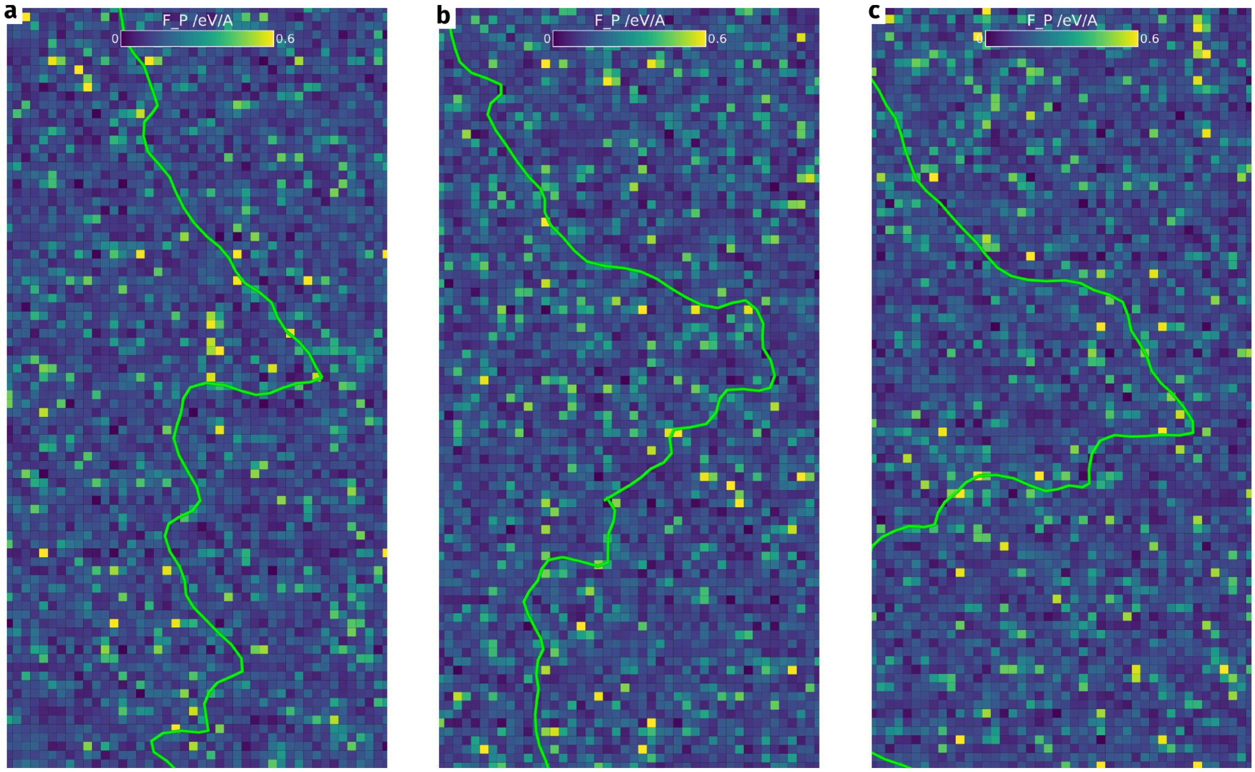

Supplementary Figure 10. **Dislocation pinning of the leading partial in the Cantor alloy.** Dislocation lines (green) during glide under constant load in the Cantor alloy superimposed on the local pinning point strength (F_P is denoted as F_P in the color bar). Here, F_P is averaged in the two lattice planes adjacent to the dislocation glide plane on a 3x3 Å grid. **a-c** The snapshots are taken at different times during the simulation. They correspond to 116.04 ps, 125.34 ps, and 112.65 ps, respectively. Note, that each images shows a different section of the dislocation line. Good agreement between observed pinning and proposed pinning points can be seen (red circles) in all three frames.

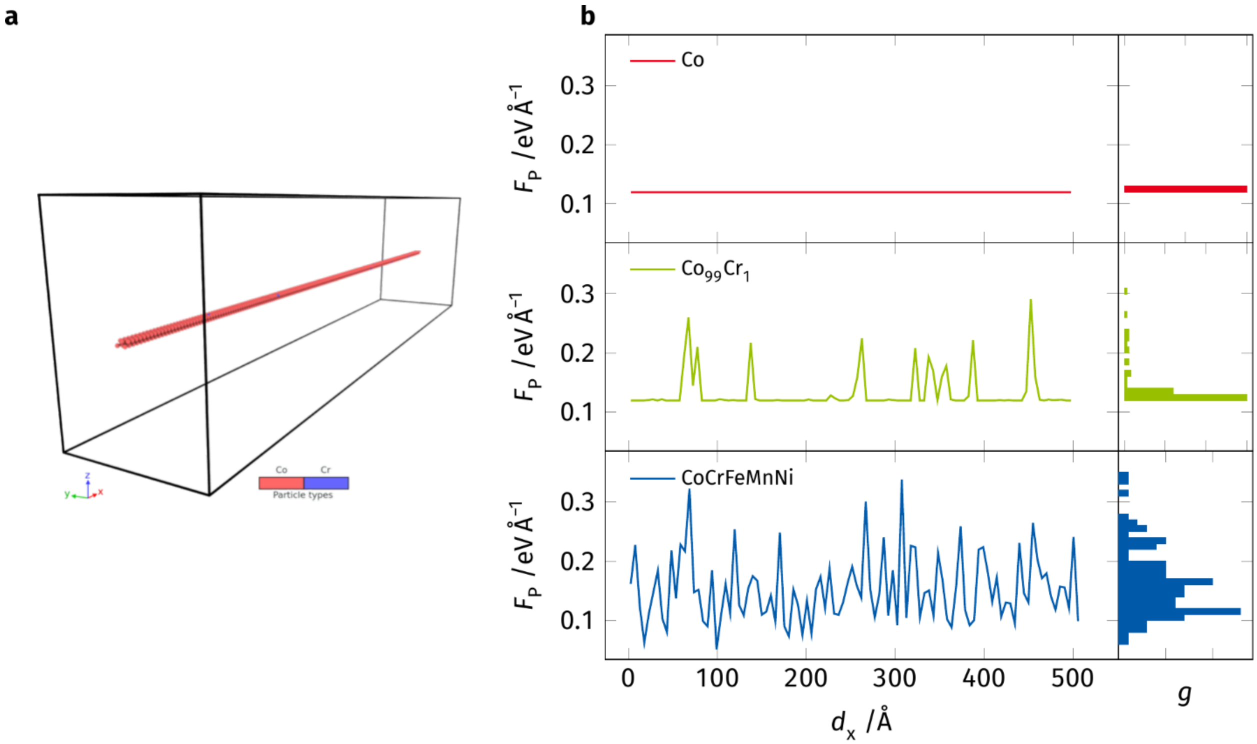

Supplementary Figure 11. **Local friction landscape encountered by a dislocation line as it moves through a pure metal, a dilute solid solution, and a concentrated high-entropy alloy.** **a** Column of atoms above and below the GSF plane measuring 6 atoms across used in the subsequent analysis. The GSF plane (normal to the z-direction) is located between these two rows. Here, the $Co_{99}Cr_1$ alloy is shown as an example as we expect a strong interaction of Co and Cr atoms based on our results shown in Fig.4. **b** Average F_P values over 100 spatial bins along the x-direction (parallel to the Burgers vector, normal to the dislocation line direction) in Co, $Co_{99}Cr_1$, and CoCrFeMnNi. On the right the distribution g of F_P in each sample is shown. In essence, there are no strong pinning points in the pure Co metal, some discrete strong pinning sites in the dilute $Co_{99}Cr_1$ solid solution and an abundance of pinning spots in the full HEA. The strong pinning spots in the CoCrFeMnNi alloy do not only show an increase in frequency but also higher peak F_P values. The data confirms the schematic representation given in Fig.5 of the main manuscript. Note that the F_P data cannot be integrated to derive the corresponding energy landscape due to the insufficient spatial resolution.

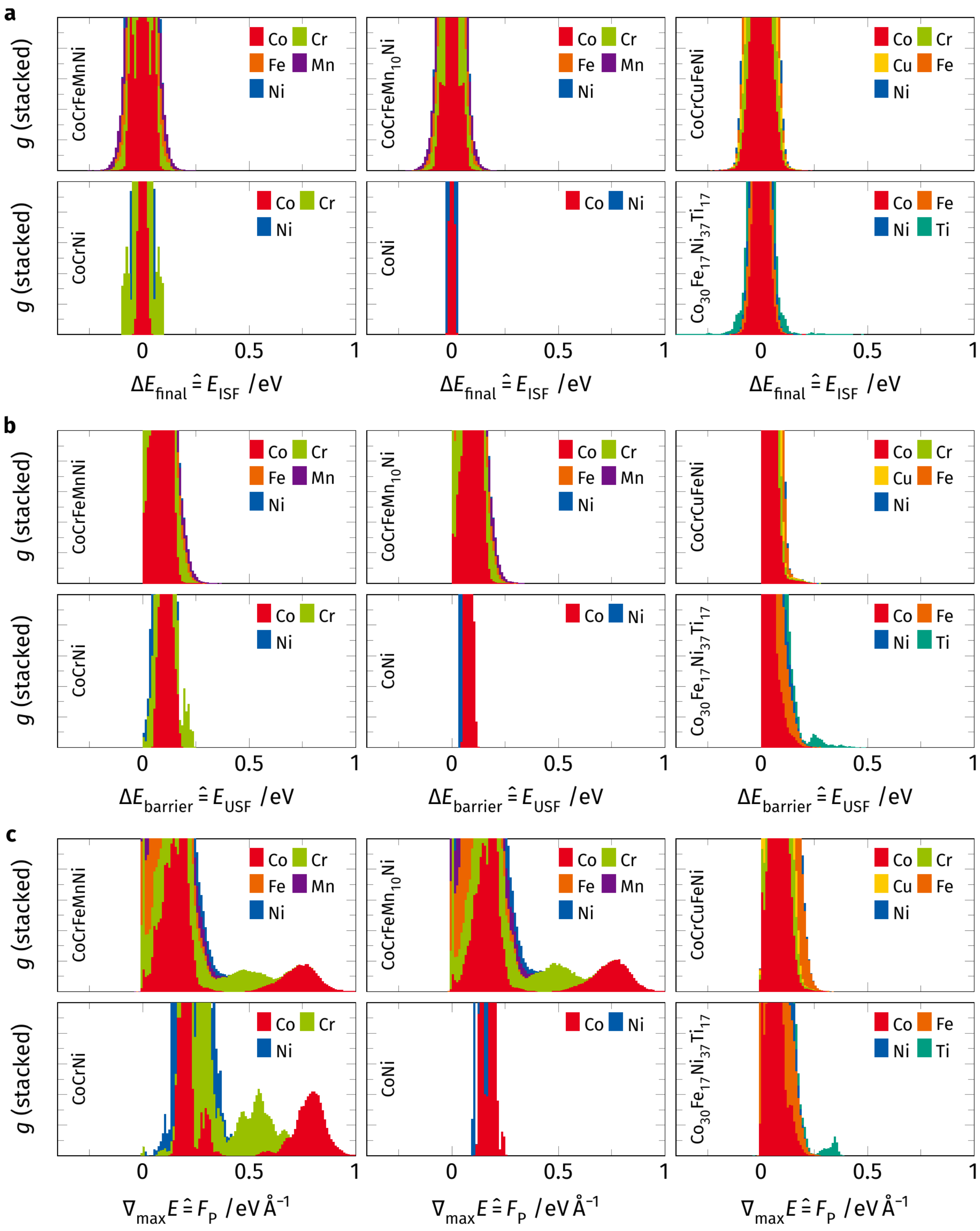

Supplementary Figure 12. **Different descriptors that might be considered for the local dislocation pinning strength.** Here we compare: F_P **a**, the stable SFE (labeled ΔE_{final} as it is the energy difference between initial and final state), **b** the unstable SFE (labeled $\Delta E_{Barrier}$ as it is the height of the energy barrier), and **c** for different material systems. All data can be extracted from the per atom energy landscapes calculated for Fig.3 of the main manuscript. The data representation corresponds to Fig.4a.

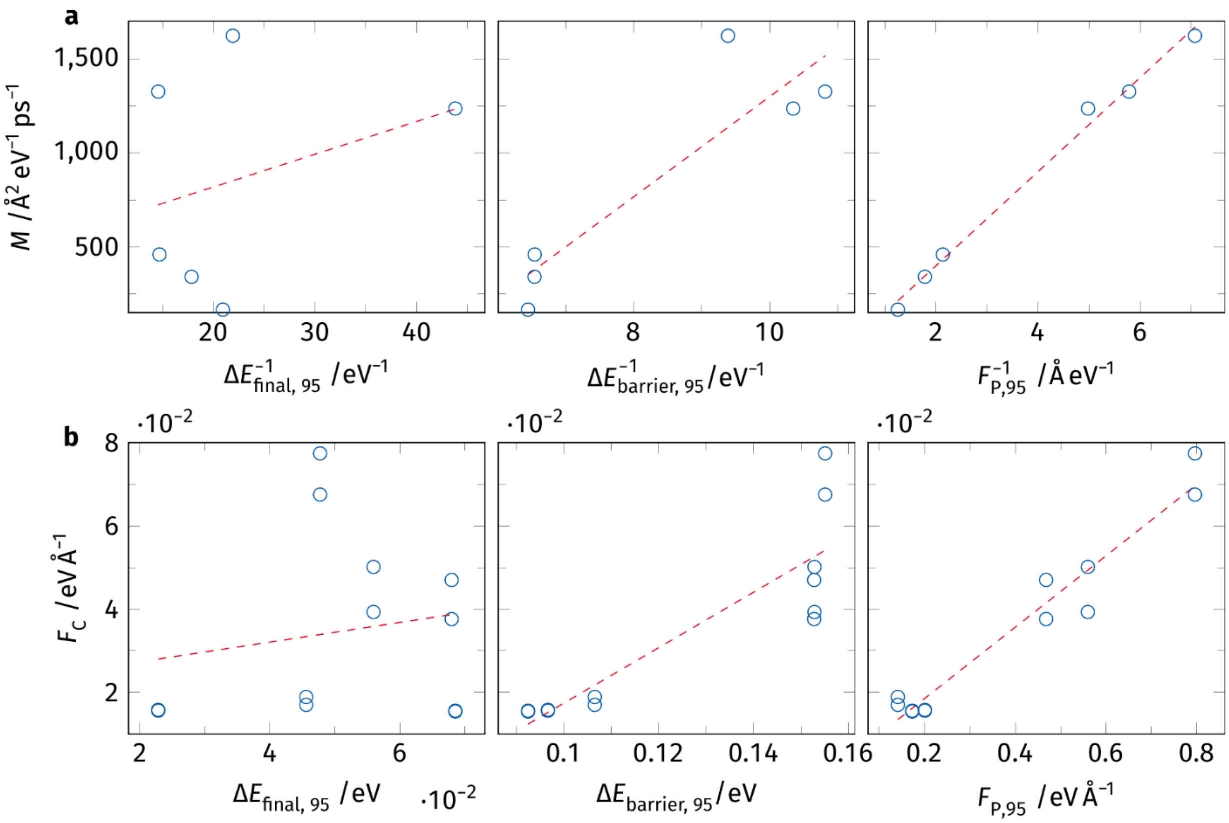

Supplementary Figure 13. **Relation of the critical force and dislocation mobility measured in all samples to the different atomic descriptors.** Correlation of the 95th percentile of each descriptor shown in Supplementary Figure 12 and discussed in the main manuscript with respect to the dislocation mobility M, **a** and the critical force F_C **b** required to initiate dislocation glide. The red line corresponds to the best linear fit through the data extracted from the simulations (marks). Best correlation can be seen for the descriptor based on F_P.

Supplementary Methods

Step by step instructions to calculate the per-atom GSF curves

- Prerequisites:
  - Establish the equilibrium lattice constant of desired composition at 0 K
- Sample preparation:
  - {111} plane aligned with one of the cartesian directions (in our case *z*-axis)
  - All atoms on ideal FCC lattice sites with the equilibrium lattice spacing
  - Alloy composition and atomic ordering as desired
- Simulation setup:
  - Define 2 groups of atoms one above and one below the GSF {111} plane
  - Periodic boundary conditions in the GSF plane, open boundaries normal to the GSF plane
- Simulation:
  - Scan atoms of group 1 across group 2, i.e., displace them in *x*- and *y*-direction (assuming the *z*-direction is {111} aligned). The scan direction / area depends on the Burges vector on the dislocation of interest.
  - Relax the system at each displacement grid point and calculate the energy. For relaxation, the forces in *x*- and *y*- direction (in the GSF plane) are set to 0, while the forces in *z*-direction (normal to the GSF plane) are averaged across all atoms in each group, respectively. This results in a rigid body displacement of the two regions (above and below the GSF plane) against each other.
  - Store the energy after each minimization (at each grid point) for each atom.
- Post processing:
  - Collect the displacement-energy maps for each atom in the GSF plane
  - Apply interpolation using, e.g., bicubic splining
  - Find the local minima (cf. Fig. 3) corresponding to the (meta-)stable lattice positions and connect them using straight lines.
  - Extract maximum gradients along these profiles
