# Supplementary material for: The origin of jerky dislocation motion in high-entropy alloys
Source: Nat Commun. 2022 Aug 15;13:4777. doi: 10.1038/s41467-022-32134-1 (PMC9378647; doi:10.1038/s41467-022-32134-1)
Supplement: Supplementary file 2 — Description of Additional Supplementary Files [file 41467_2022_32134_MOESM2_ESM.pdf]

Description of Additional Supplementary Files

File Name: Supplementary Movie 1
Description: Movie 1 shows the glide of a series of partial dislocations during in-situ TEM straining. To increase the signal-to-noise ratio by removing the background features, the last frame is subtracted from all other frames after aligning the frame by using cross-correlation drift correction. The dark bands in the video are stacking faults of which left and right ends are trailing and leading partial dislocations, respectively.

File Name: Supplementary Movie 2
Description: Movie 2 shows the leading partial dislocation (red line) under applied constant shear load migrating through the chemically complex HEA matrix. The underlying "landscape" corresponds to the. The per-atom pinning point force FP values are averaged on a 3 Å by 3 Å grid and smoothed using Gaussians for visual representation. It corresponds to the sequence shown in Fig. 3 c
